# Effect of Dissection and Reconstruction of Palatal Muscles on Morphological Features and Ultrastructure of the Oral Musculature in Cats

**DOI:** 10.1155/2016/6807678

**Published:** 2016-09-06

**Authors:** Wei Han, Zhiyong Wang, Xiaofeng Qi, Wenguang Xu, Hao Shen, Bing Shi, Yong Lu

**Affiliations:** ^1^Department of Oral and Maxillofacial Surgery, Nanjing Stomatological Hospital, Medical School of Nanjing University, Nanjing, China; ^2^State Key Laboratory of Oral Diseases, Department of Cleft Lip and Palate Surgery, West China Hospital of Stomatology, Sichuan University, Chengdu, China

## Abstract

The study was designed to determine the effect of dissection and reconstruction of palatal muscles on muscle morphology in cats. 27 cats were randomly divided into three groups according to the extent of muscle dissection from the palatal midline. All dissections were performed from the posterior border of the hard palate, and the muscles were allowed to reconstruct over time. The morphological features were determined by hematoxylin and eosin staining of tissue sections, and ultrastructure was observed under a transmission electron microscope. As a result, no obvious differences were evident in the morphological features or ultrastructure of animals in the <1/3rd and 1/3rd-2/3rd area groups. In the >2/3rd area group, the muscles fibers were disordered and inflammatory cell infiltration and naïve muscle cells were found at one month after surgery. At the second and third month after surgery, the muscle fibers showed regular alignment, the naïve muscle fibers gradually matured, and the number of infiltrating inflammatory cells decreased. Muscle ultrastructure analysis revealed that myocommata were correctly aligned, and the Z line was more distinct. In conclusion, extensive dissection of palatal muscles does not result in fibrosis. Injury to oral musculature can be repaired and the musculature regenerated over time.

## 1. Introduction

The levator veli palatine (LVP) is the most important muscle involved in the movement of the soft palate. Velopharyngeal closure depends on good motor function of the LVP. During cleft palate repair, recovering the LVP to its normal anatomical position by dissecting and retreating the correct end and rebuilding levator support is of vital importance for achieving optimal voice recovery after surgery [1–4]. Whether the dissection and separation of palatal muscles during surgery result in muscle fibrosis that can affect the movement of the soft palate and whether a relationship exists between the scope and extent of anatomy and the repair of muscle injury have hitherto not been investigated [5, 6]. Here, we dissected, separated, reset, and rebuilt the palate muscles of cats to varying degrees in order to simulate clinical cleft palate repair and determined its success in the functional reconstruction and structural organization of the LVP and the soft palate so as to obtain a theoretical reference point for clinical application.

## 2. Materials and Methods

### 2.1. Ethical Approval

All the experimental procedures were conducted in full accordance with the National Institutes of Health Guide for the Care and Use of Laboratory Animals and were approved (approval number: 2015NL-006(KS)) by the Ethics Committee of Nanjing Stomatological Hospital, Medical School of Nanjing University. Full measures were taken to minimize animal discomfort or pain during the experiments.

### 2.2. Experimental Animals and Grouping

**Figure 1:**
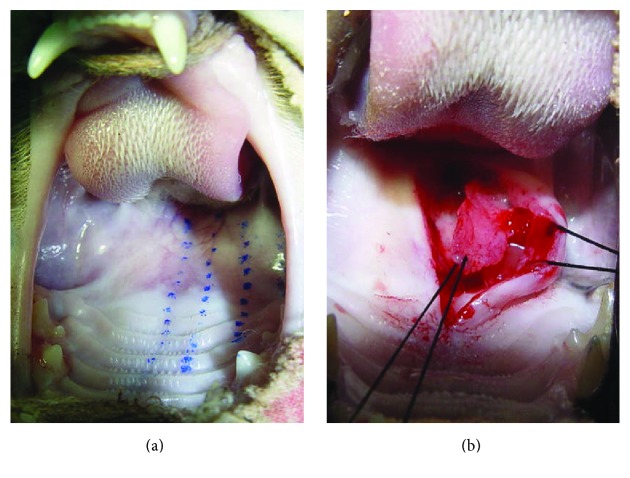
Schematic diagram of soft palate muscle dissection in cat and the dissected soft palate muscle.

We selected 27 male, healthy, and vibrant domestic cats (*Felis catus*) aged 12–24 months and weighing 1.5–2.5 kg. The cats were reared in a controlled environment at 18–25°C with 40%–70% relative humidity. The soft palate, with the border from the hamular process of one side to the palatal midline, was equally divided into three parts, indicative of the different extents and scopes of palatal muscle dissection. The animals were divided into three groups depending on the anatomical extension of the muscles. Group 1 corresponded to the anatomical region of muscles from the palatal midline to the first one-third (≤1/3rd) area of the hamular process. Group 2 corresponded to the anatomical region of muscles from the palatal midline to second one-third (1/3rd-2/3rd) area of the hamular process, and Group 3 corresponded to the anatomical region of muscles from the palatal midline to the area beyond 2/3rd of the hamular process. The 27 cats were randomized into three groups; the palatal muscles on the other side of the palatal midline served as the control group in all animals (Figure 1(a)).

### 2.3. Preparation for Animal Models

All animals were starved for 4 h before surgery. After being anesthetized by an intramuscular injection of 0.5 mg/kg sumicanxin, the animal was disinfected and spread on towels. The oral cavity was exposed, and the stratum mucosum of the palatal midline was excised so that the anterior extremity extended to the posterior border of the hard palate and the posterior extremity to the end of the soft palate. After separating and exposing the oral mucosa with a small periosteal separator, the muscle layer composed of the palatal muscles attached to the aponeurosis palatina of the posterior border of the hard palate could be visible. The aponeurosis palatina attachment was cut without pricking the nasal mucosa and then completely dissected and separated from the nasal mucosa. The dissection should be continued until the nasal stratum mucosum of the nose appears “blue,” which indicates that the separation of muscles is complete. Depending on the experimental group of each animal, an apposition suture with a size of 3-0 nonabsorption line was used to connect the amputated muscle stump with the contralateral side of the muscle, and the oral mucosal layer was closed with an interrupted suture (Figure 1(b)). After the surgery, each cat was fed liquid diet for one week, and intramuscular penicillin injections (0.2 million units/day) were administered for three consecutive days.

### 2.4. Immunohistochemical Analysis

Animals were sacrificed by venous air embolism at one, two, or three months after surgery. Soft palatal muscle specimens of size 1.5 cm × 0.5 cm × 0.3 cm were excised from the experimental side and then sliced equally into two slices. One slice of each animal was fixed in 10% formalin solution. After conventional dehydration and paraffin embedding, approximately 3 *μ*m thick serial sections were obtained and stained with hematoxylin and eosin. The muscle tissue structures were then observed under light microscope. The second slice was immediately transferred to 2.5% glutaraldehyde fixation solution and maintained for 2 h. The slice was then flushed with distilled water, dehydrated in 50%–100% acetone, and embedded in Epon812. The slice was sectioned using ultramicrotomy into 30 consecutive layers, double stained with uranium-lead, and observed under a Hitachi-600 transmission electron microscope. The soft palate muscles of the other normal side were also treated as described above and used as the control group.

## 3. Results

The palatal wounds were free of infections or dehiscences and healed spontaneously over time.

### 3.1. Histological Findings

**Figure 2:**
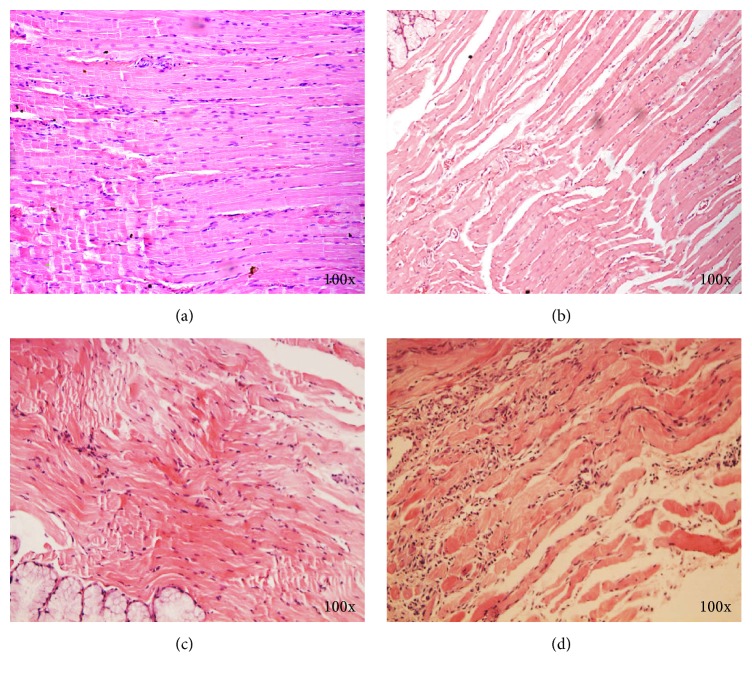
Histological results of 3 different groups at different stages. The muscle fiber alignment was regular and dense, with clear and distinct cross striations 3 months after surgery in Group 1 (a). The muscle fiber alignment was regular and loose 1 month after surgery in Group 2 (b). The arrangement of muscle fibers appeared regular without infiltration of inflammatory cells or formation of collagen deposits 3 months after surgery in Group 2 (c). The cell nuclei appear in the middle and nucleoli were distinguishable 3 months after surgery in Group 3 (d).

In Group 1, the muscle fibers were comparatively neat, loose, without fracture, and free of inflammatory cells one month after the surgery. At two months after surgery, the muscle fibers were aligned and densified but did not show collagen deposits. At three months after surgery, the muscle fibers were completely aligned with clear and regular transverse striations, whereas the outer membrane was without fiber hyperplasia (Figure 2(a)).

In Group 2, the muscle fibers were slightly disarranged with minor tears, but without inflammatory cell infiltration at one month after surgery (Figure 2(b)). At two months, muscle fibers were comparatively aligned, without infiltration of muscle giant cells or inflammatory cells. At three months, the muscle fibers were uniform and completely aligned, with clear transverse striations, and free of inflammatory cell infiltrates (Figure 2(c)).

In Group 3, the muscle fibers were neatly aligned and consisted of some intermittent naïve muscle fibers but were without inflammatory cell infiltrates. At two months, the naïve muscle fibers had relatively matured, and their nuclei shifted toward one side and did not show any clear karyosomes. At three months, the fibers were completely aligned and free of inflammatory cell infiltrates or fiber hyperplasia. The parazone and silent zones were distinctly visible, and no obvious connective tissue formation was observed in the muscles (Figure 2(d)).

In the control group, the fibers were completely aligned and densified with clear transverse striations at all months.

### 3.2. Structural Reorganization

**Figure 3:**
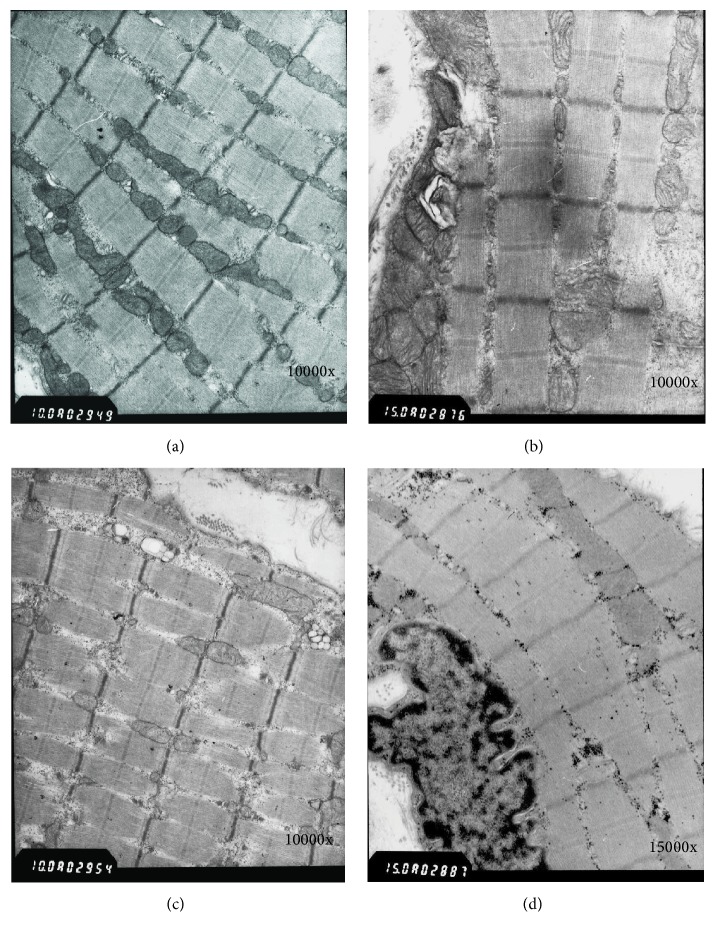
Hitachi-600 transmission electron microscopes of 3 different groups at different stages. Myofibrils were aligned, Z lines were distinct, and myocommata were of uniform length 1 month after surgery in Group 1 (a). Very few mitochondria were oncotic and unmedullated and myocommata were aligned 3 months after surgery in Group 2 (b). Myocommata were sparsely arranged 1 month after surgery in Group 3 (c). Myocommata were aligned and glycogen content increased 2 months after surgery in Group 3 (d).

In Group 1, the length of the muscle segment was even, and the Z line was clear at one month after surgery. The endoplasmic reticulum appeared slightly expanded in some cells, and the number of mitochondria increased slightly (Figure 3(a)). At two months, the mitochondria appeared normal in size and structure and were well distributed. At three months, both Z line and muscle fibers were correctly aligned.

In Group 2, the Z line was in order, with increased number of mitochondria. The sarcoplasmic reticulum was expanded. At two months, there was an increase in the number of sarcoplasmic reticulum tubules; the mitochondria appeared oncotic and had increased in number. Moreover, the muscle fibers were aligned, and the Z line was clear. At three months, the number of oncotic mitochondria had decreased, but none exhibited signs of pulpy disease. The muscle fibers were completely aligned, and the Z line was clear and distinct (Figure 3(b)).

In Group 3, at one month, the sarcomeres were slightly crumbled, and the Z line was fuzzy. The muscle fibers were narrow and grew downwards, and the number of mitochondrial cristae was reduced (Figure 3(c)). At two months, the density of the mitochondrial cristae slightly increased, the muscle segment was correctly aligned, and more glycogen deposits were present. The muscle cell nuclei and the muscular mantle were normal (Figure 3(d)). At three months, the number of mitochondria increased, and sarcomeres were aligned. The Z line was clear, and the structure of the sarcolemma was normal.

In the control group, the Z line of muscle fibers was clear and distinct, the nuclei were normal, chromatin was well distributed, and the mitochondrial cristae were normal.

## 4. Discussion

Experimental animal models of congenital cleft lip and palate reconstruction have been developed in mice to study the etiology and pathogenesis of the teratogenic potential of corticosteroids and vitamin A [7–9]. We chose the cat as our experimental model for cleft lip and palate studies because cats being carnivorous have a large oral fissure, are easy to operate and observe, and are cheaper to raise than dogs or primates [10, 11].

We adopted a model not involving fracture in this study and attempted to eliminate all potential confounding factors by simulating the effect of dissection and repositioning of the soft palate muscles on the soft palate structure via the paleognathous operation. The effects of the repositioning on the histological features of soft palate muscles were then determined.

Most researchers recommend that the soft palate muscles should be recovered and reconstructed during cleft palate repair in order to achieve effective velopharyngeal closure. However, a few researchers believe that changes to the muscle anatomy and detachment resulting from reorganization may damage the surrounding blood vessels, thereby resulting in muscle fibrosis and affecting the movement function of the soft palate [12, 13]. Our study findings show that detachment of the first 1/3rd region from the palatal midline does not affect the histological structure of the soft palate muscle. When the middle area (2/3rd area from the palatal midline) was excised, the muscle tissue was slightly damaged and the repair time increased. However, the muscle fiber was aligned and had pyknotic nuclei, without fibrous connective tissue or inflammatory cell infiltration. When the anatomical scope was the largest (i.e., when >2/3rd of the region from the palatal midline was excised), muscle fibers were disordered and nonpyknotic in the early stages after surgery. However, a fractured appearance was evident, and cytopoiesis occurred as detected by the presence of immature naïve muscle fibers. However, the muscle fibers were correctly aligned and pyknotic and the naïve muscle fibers tended to mature 2-3 months after surgery. Thus, our results show that when the anatomical scope is large, the degree of muscle damage and time required for restoration of histological features of muscles to their normal appearance increase.

Mitochondria are one of the most sensitive indicators of cell damage. Intracellular changes in the morphological and structural features of mitochondria can help determine the state of soft palate muscle repair after anatomy and separation [14, 15]. Ultrastructural findings of our study show that when the anatomical scope was minimal (Group 1), no abnormal changes occurred in the structure of the mitochondria regardless of the stage of repair. When the anatomical scope was comparatively larger (Group 2), there was an increase in the number of mitochondria and an expansion of the sarcoplasmic reticulum. Moreover, the structural features and number of mitochondria were normalized during the late repair stage. When the anatomical scope was the largest (Group 3), the mitochondrial cristae number and glycogen metabolic energy demand increased, and muscle fibers were not aligned or pyknotic during the early postoperative stage. However, abnormal pathological changes, such as decreased size and number of mitochondrial cristae and changes in mitochondria vacuoles, were restored at three months after the surgery. Moreover, the structure of mitochondria had returned to a comparatively normal state, the structure of nuclei and muscle membranes had normalized, and no abnormalities were observed in the surrounding vessel walls at this time point. Thus, our study results demonstrate that separation of a large region from the palatal midline causes some damage to the muscle tissue, and an aggravation of cellular functions increases the number of mitochondrial cristae, but no pathological changes in muscle ultrastructure occur.

Skeletal muscle regeneration is usually poor and depends on several factors, such as fibrosis of the scar tissue, restoration of blood circulation within the damaged area, and extent of retention of the muscle membrane [16–18]. The surgery performed in our study simulates the clinically used LVP reconstruction proposed by Sommerlad [14, 19]. Here, we dissected, separated, and repositioned the soft palate muscle to varying degrees from the trailing edge of the hard palate. The surgery resulted in complete separation of the nasal mucosa rather than dissection of the muscle, which helped in not only retaining the integrity of the muscle tissue, but also reducing injury to the surrounding blood vessels [4, 19]. We recommend dissection of an area larger than two-thirds the area from the palatal midline because the damage to muscles and blood supply was not considerably large. The histological and ultrastructural findings confirmed that although this procedure caused mild damage to the muscles, no pathological changes with regard to muscle membranes or nuclei were observed. In fact, the repair process mainly involved the formation of naïve muscle cells, which tended to mature over time and help in restoration of the muscle structure.

In the present study, we successfully simulated clinical cleft palate repair and evaluated the effect of functional reconstruction and structural organization. However, there are still some doubts that remain to be settled. One notable point was the relationship between dissection degree of soft palate muscles and nerve injury in cleft palate repair. As known to all, the movement of the soft palate muscles plays a pivotal role in velopharyngeal closure. So far, most studies have focused on the anatomical repair of the musculature of the cleft palate [20]. However, the possible nerve injury resulting from the surgical dissection was scarcely mentioned in surgical techniques guidelines. Furthermore, after a careful review of related literatures, we found that it remained inconclusive in which nerve innervates soft palate muscles [21]. Only a few studies investigated the innervations of the soft palate muscles in humans. Shimokawa et al. discovered that levator veli palatine (LVP) was dually innervated by lesser palatine nerve (LPN) and pharyngeal plexus (PP) [20]. This review of the literature demonstrates the lack of accurate information about the innervation of the LVP and palatopharyngeus muscle. Most likely, the LPN and the PP dually innervate these two muscles. Anatomical studies illustrate that PP is located on the lateral surface of superior pharyngeal constrictor muscle. At the front, its branches run through superior pharyngeal constrictor muscle and enter the LVP and finally penetrate the back surface of LVP innervating upper part of LVP. At the back, branches of PP innervate inferior pharyngeal constrictor muscle and middle pharyngeal constrictor muscle and then extend as a lot of fine branches, most of which pass into LVP. The LPN runs through the lesser palatine foramen and runs over the palatine aponeurosis of the TVP and the nasal part of the PP to enter the inferior-velar part of the LVP on its lateral surface. Anatomical studies suggest that, during surgical dissection, caution should be taken to dissect the dorsal/lateral aspect of the LVP from the PP because that is the region where the LPN enters the LVP [20, 22, 23]. In addition, some anatomical studies have illustrated that if the soft palate muscles were dissected appressed by the nasal side of muscle and the outer lateral side of soft palate muscles was not involved in the dissection region, the LPN would not be injured [22]. Similarly, it is the same with Group 3 which corresponded to the anatomical region of muscles from the palatal midline to the area beyond 2/3rd of the hamular process in our study. However, in view of complicated anatomical structure and inconclusive innervation of soft palate muscles, more researches may need to be conducted in the future to further investigate the relationship between the dissection degree of soft palate muscles and nerve injury in soft palate repair.
